# Effects of Supplemental Effective Microorganisms in Feed on the Growth, Lipid Deposition and GH–IGF‐I Axis of the Blunt Snout Bream *Megalobrama amblycephala*

**DOI:** 10.1155/anu/5564422

**Published:** 2026-02-06

**Authors:** Hao Zhu, Fan Wu, Xuan Che, Jinxing Xiao

**Affiliations:** ^1^ Fishery Machinery and Instrument Research Institute, Chinese Academy of Fishery Sciences, No. 63 Chifeng Road, Yangpu District, Shanghai, 200092, China, cafs.ac.cn; ^2^ Key Laboratory of Aquaculture Facilities Engineering, Ministry of Agriculture and Rural Affairs, Shanghai, 200092, China, agri.gov.cn; ^3^ Ocean Research Center of Zhoushan, Zhejiang University, No. 10 Lincheng Tiyu Road, Zhoushan, 316021, Zhejiang, China, zju.edu.cn

**Keywords:** digestive enzymes, EM, GH, IGF-1, liver, *Megalobrama amblycephala*

## Abstract

The aim of this study was to investigate the effects of an effective microorganism (EM)‐based microbial inoculum on the growth and expression of related genes in 1‐year‐old healthy *Megalobrama amblycephala*. The bacterial strains included *Lactobacillus plantarum*, *Lactobacillus fermentum*, *Enterococcus faecium*, *Bacillus velezensis*, *Paenibacillus polymyxa* and *Lactococcus* sp. The fish were randomly divided into five groups: one group was fed a basal diet (control group), and the other groups were provided feed containing EMs at levels of 5, 10, 20 or 40 g/kg. The growth performance, gastrointestinal digestive enzyme activity and expression of the growth hormone (GH), GH receptor A (GHRA), GH receptor B (GHRB) and insulin‐like growth factor‐1 (IGF‐1) genes in the liver of *M. amblycephala* after 60 days of outdoor cage culture were analysed. The weight gain rate for *M. amblycephala* significantly increased when EM was added to the feed, and the 10 g/kg EM group had the greatest weight gain, which was 7.34% higher than that of the control group; this group also presented the highest intestinal protease and lipase activities. Regarding hormone levels, GH, GHRA, GHRB and IGF‐1 expression levels were highest in the 10 g/kg EM group. Additionally, EM addition significantly improved lipoprotein metabolism in the liver, resulting in a decrease in low‐density lipoprotein levels and an increase in high‐density lipoprotein levels. The results of this study suggest that EM supplementation in feed improves the growth performance of *M. amblycephala*, reduces the risk of fatty liver and affects the expression of growth genes.

## 1. Introduction

The rapid development of intensive aquaculture has led to an increasing demand for high‐efficiency and low‐cost feed. Fish feed is subject to high protein requirements, which also leads to an increase in feed costs. Therefore, appropriately incorporating non‐protein energy sources can reduce feed costs and improve protein use. The addition of non‐protein energy sources can also cause hepatic fat accumulation in fish, leading to disease. Research and development of additives that promote growth and reduce fat deposition have become hot research topics.

Effective microorganisms (EMs) constitute a compound microbial preparation that comprises more than 80 microorganism species belonging to 10 genera, such as *Lactobacillus*, photosynthetic bacteria, actinomycetes and yeast. EMs benefit aquatic environments and ecosystems [1–3]. Their components, for example, lactic acid bacteria, phototrophic bacteria, actinomycetes and yeasts, can be used to address a wide suite of waste‐water problems, such as the bioremediation of heavy metals and the prevention of eutrophication [4, 5]. EMs can not only inhibit the proliferation of pathogenic bacteria but also promote growth by forming dominant flora in the intestine to degrade macromolecular nutrients [6–9], indicating that they have potential applications in species such as tilapia (*Oreochromis niloticus*) and common carp (*Cyprinus carpio*) [10–13]. However, species differences in the regulation and molecular mechanisms of lipid metabolism still exist.

Lipid metabolism in fish is centred on triglycerides (TGs) and cholesterol (CH), and the levels of TG and CH deposited in tissue directly reflect the health status of the liver [14]. Moreover, the activities of digestive enzymes such as intestinal protease and lipase are key indicators for evaluating nutrient utilisation efficiency [15, 16]. Furthermore, the nutritional status has been demonstrated to be the principal regulator of the growth hormone (GH)–insulin‐like growth factor‐1 (IGF‐I) axis [17, 18]. Li et al. [19] reported that an improvement in intestinal digestion in the blunt snout bream affects IGF‐I expression in the liver. Therefore, investigations of this axis might be helpful for revealing the correlations between effective dietary microorganisms and the growth performance of fish.

*Megalobrama amblycephala* is an important herbivorous economic fish in China that is widely cultivated because of its excellent growth performance and high stress resistance [20–22]. In this study, the effects of EM bacteria on digestive enzyme activity, lipoprotein metabolism and the expression of genes involved in the GH/IGF axis in *M. amblycephala* were analysed, and their growth‐promoting effects and metabolic safety were systematically evaluated, providing a theoretical basis for revealing the molecular mechanism through which microecological agents regulate fish growth and helping us choose suitable additives to promote efficient growth.

## 2. Materials and Methods

### 2.1. Experimental Fish and Diets

The animal experiments were conducted at Xinwu Town Experimental Base (30°57′02″N, 121°09′03″E) in Songjiang district, Shanghai city, from 5 August to 4 October 2024. *M. amblycephala* were obtained from the pond at the base. Before the experiment, *M. amblycephala* were temporarily reared in a 5000 m^2^ soil pond for 15 days and fed daily with freshwater fish compound feed (with a crude protein content of 28.23%) (Table 1). Dry EM powder was purchased from the Zhoushan Ocean Research Center of Zhejiang University. The EM powder included *Lactobacillus plantarum*, *Lactobacillus fermentum*, *Enterococcus faecium*, *Bacillus velezensis*, *Paenibacillus polymyxa* and *Lactococcus* sp. (Table 2), with an effective viable bacteria count of 10^11^ CFUs/g. The formula for the culture medium was 20 kg of water, 1 kg of brown sugar and 0.1 kg of urea. A total of 20 g of EM powder was dissolved in 20 kg of water, after which 1 kg of brown sugar and 0.10 kg of urea were added, followed by shaking to ensure even mixing. The culture medium was dissolved and cooled. The EMs were then cultured for 2 days without stirring at 30–35°C. When the pH decreased to ~4, the EM solution was considered prepared and subsequently stored at 4°C.

**Table 1:** Formulation and proximate composition of the basal diet.

Ingredients	%
Fish meal	4.00
Soybean meal	8.50
Rapeseed meal	24.00
Cottonseed meal	22.00
Wheat bran	11.00
Wheat middlings	20.00
Rice bran	6.00
Calcium biphosphate	2.00
Soybean oil	1.50
Premix	1.00

Proximate composition	
Crude protein	28.23
Crude lipid	5.88
Crude fibre	7.23
Ash	6.67
Total phosphorus	1.01
Gross energy (MJ/kg)	18.44

**Table 2:** Percentage of the community abundance at the genus level in the fermentation process.

Name	Time (%)				
	1	2	4	8	16
*Lactobacillus plantarum*	5.23	6.06	14.37	32.26	69.98
*Acetobacter tropicalis*	0.06	8.99	26.48	11.22	0.01
*Acinetobacter pittii*	10.12	10.20	3.80	1.00	0.00
*Lactobacillus fermentum*	18.12	20.20	13.08	20.84	30.00
*Enterococcus faecium*	15.17	22.31	10.68	6.64	0.00
*Bacillus velezensis*	20.14	14.83	5.09	4.13	0.00
*Unclassified_f__Enterbacteriaceae*	17.89	0.53	0.36	5.18	0.00
*Pseudomonas*	1.01	3.88	1.13	0.11	0.01
*Paenibacillus polymyxa*	6.13	5.77	10.79	5.58	0.00
*Lactococcus* sp.	5.13	5.23	10.23	5.04	0.00

*Note:* The solution containing effective microorganisms was directly sprayed onto the feed, which was fed to the fish to prevent mould growth in the feed.

The preparation of the culture medium was based on the requirements for carbon and nitrogen sources for microbial growth, and brown sugar and urea were selected as the main raw materials for the culture medium.

Feeding trials were conducted using small cages (2.00 × 2.00 × 1.50 m). Fifteen small cages were fixed in the middle of each pond (30 × 20 m), and at least two microporous aerators were placed in each cage. The depth of the pond was 1.50 m, and the effective depth was 1.20 m. The 15 breeding cages were fixed in the pond with stakes and bottom anchors. A temporary purse seine area far from the cages was established to stock *M. amblycephala* fry to supplement fish that died in the breeding process.

### 2.2. Experimental Design and Breeding Trial

Different volumes of the EM solution (5, 10, 20 and 40 g of bacterial mixture per kg of feed) were directly and evenly sprayed onto the feed before each feeding process. A total of 450 healthy *M. amblycephala* fingerlings (175.00 ± 12.00 g) with the same specifications were domesticated for 2 weeks and randomly divided into 5 groups, with 3 replicates per group (i.e., 15 small cages) and 30 fingerlings in each cage. The fish were fed the experimental feed for 60 days. During the experiment, the animals were fed at 07:00, 12:00 and 17:00 daily, and the feeding quantity was recorded. After 30 min, the feeding conditions were observed and recorded. During the experiment, the pH of the water was maintained between 7.00 and 8.00, and the dissolved oxygen content was greater than 5.00 mg/L. The ammonia nitrogen concentration was maintained at less than 0.40 mg/L, and the nitrite concentration was maintained at less than 0.02 mg/L to ensure excellent water quality.

On the 8th day of compound probiotic fermentation, the richness of the microbial community was the highest, and with increasing fermentation time, the microbial community structure differed. Among the components of the microbial community, Firmicutes is the main dominant flora, mainly including *Lactobacillus plantarum*, *Acetobacter tropicalis*, *Acinetobacter pittii*, *Lactobacillus fermentum*, *Enterococcus faecium*, *Bacillus velezensis*, *Unclassified_f__Enterbacteriaceae*, *Pseudomonas*, *Paenibacillus polymyxa* and *Lactococcus* sp.. The abundance of other strains was relatively low, and some strains have not been identified. Useful bacterial strains included *Lactobacillus plantarum*, *Lactobacillus fermentum*, *Enterococcus faecium*, *Bacillus velezensis*, *Paenibacillus polymyxa* and *Lactococcus* sp.

### 2.3. Sample Collection

After the feeding experiment, the fish were not fed for 24 h, and the fish in each tank were weighed. Three fish with similar weights were selected from each tank. Two millilitres of blood was collected from the tail vein of one of the three fish. After the blood sample was allowed to stand for 30 min, the serum was centrifuged at 4°C (1500 rcf and 10 min). The other two fish were euthanised with anaesthetics, the abdominal cavity was opened and the hepatopancreas was removed, weighed and placed in a 2.0 mL centrifuge tube. Then, all other internal organs in the abdominal cavity were harvested. After descaling, the dorsal muscle was harvested, deboned and placed in a self‐sealing bag. The entire process was performed at low temperature (4°C). Serum, liver, pancreas and muscle samples were collected and stored at −20°C.

### 2.4. Calculations and Statistical Analysis

After the feeding trial, feeding was stopped for 1 day. The number of *M. amblycephala* was counted, and the weight gain rate and feed coefficient for the experimental group and the control group were calculated using the following formulas:

Weight gain rate=final average body mass–initial average body mass/initial average body mass×100%,

Condition factor=100×body weight/body length3,

HSI hepatosomatic index=liver weight/body weight×100%,

Feed coefficient ratio=total diet fed dry weight/total wet weight gain×100%,

Specific growth rate=LnWt−LnW0×100/t,

Protein efficiency ratio=final wet weight gain/crude protein fed×100%.

The total protease activity was measured with Flynn phenol reagent. Via the use of bovine serum albumin as the standard, the protein content in the serum and liver was determined using Coomassie brilliant blue. CH, TG, high‐density protein and low‐density protein levels were measured using kits from Nanjing Jiancheng Institute of Bioengineering in accordance with the manufacturer’s instructions.

Liver tissue preserved in liquid nitrogen was removed and ground in liquid nitrogen, after which total RNA was extracted with TRIzol. Subsequent experimental steps were performed in accordance with the methods described in the Prime Script 1st Strand cDNA Synthesis Kit. The quality and concentration of RNA were detected via 1.5% agarose gel electrophoresis and nanodroplet spectroscopy, respectively. RNA with an OD 260/OD 280 value between 1.9 and 2.1 was used for subsequent molecular experiments. cDNA was synthesised using a common reverse transcription kit (Takara, Japan). Primers were designed using Primer 5.0, and the cDNA sequence was obtained from GenBank (Table 3).

**Table 3:** Sequences of the primers used in this study.

Gene	Sequence (5′‐3′)	Reference or query gene ID
*LGR2-F*	TCTCCTACGCCTACCACT	MT585155.1
*LGR2-R*	GATGACCGCATACCTTCT	
*CHS-F*	CCAGGGACACCCATCTAC	KP710198.1
*CHS-R*	ATCTCAATCCGCAAGCAG	
*EcR-F*	AAATGAAGGTGGACAATG	MH459143.1
*EcR-R*	TCTCAGGTCGGTCAGGAT	
*CDAl-F*	TCTCAGGTCGGTCAGGAT	MF360010.1
*CDAl-R*	TTGTGGGAAACGAAGAAA	
*CaMK I-F*	ACAACTACCACCCTCACC	KF469224.1
*CaMK I-R*	ACAACTACCACCCTCACC	
*RXR-F*	ACCCTACAGATGAGCAAC	KC460324.1
*RXR-R*	TCTTTAACACCCATACTTC	
*β-actin-F*	TTCCCTGGAGAAGTCTTACGAG	KC460324.1
*β-actin-R*	TTGGCATACAAGTCCTTACGG	

The expression levels of GPx 1, GH, IGF‐1, β‐actin and 18S RNA were measured by polymerase chain reaction (PCR) using a SYBR Prime Script RT–PCR Kit from TaKaRa. Fluorescence quantitative PCR was conducted on a CFX96 TM real‐time system (Bio‐Rad, USA). The PCR volume was 20 μL:1 μL of cDNA template, 10 μL of SYBR, 0.5 μL of upstream primer, 0.5 μL of downstream primer and 8 μL of enzyme‐free water. After mixing and then centrifugation at low speed at 4°C for 5 min, the reaction procedure was as follows: predenaturation at 95°C for 30 s and 40 cycles of denaturation at 94°C for 5 s and annealing at 55°C for 30 s. The melting reaction conditions ranged from 65°C to 95°C, and fluorescence was recorded for 2 min [23].

The Ct values of each sample were normalised to those of β‐actin and 18S RNA, which served as internal reference genes. The relative expression of the GH and IGF‐I mRNAs in the livers of fish in the low‐, medium‐ and high‐density groups was calculated via the 2^−ΔΔCt^ method.

### 2.5. Statistical Analysis

The experimental results are presented as the means ± standard deviations (SDs). SPSS 13.0 software was used for one‐way analysis of variance (ANOVA) of the experimental data, followed by the least significant difference (LSD 0.05) test.

The data were analysed using one‐way ANOVA combined with Tukey’s multiple range tests with SPSS, Version 19. Statistically significant differences were indicated by *p* < 0.05. The values are presented as the means ± SDs (*n* = 3).

## 3. Results

### 3.1. Growth Performance

At the end of the 60‐day growth experiment, the addition of EMs to the feed significantly affected the growth characteristics of *M.amblycephala* (Table 4). The addition of EM bacteria had a significant effect on the fat percentage, liver–body weight ratio, weight gain rate and feed coefficient but had little effect on comparative growth and protein efficiency. As the concentration of EM bacteria increased from 5 to 40 g/kg, the hepatosomatic index and weight gain rate increased to varying degrees, whereas the fat rate and feed coefficient decreased to varying degrees. The weight gain rate of the 10 g/kg group was the greatest (7.34%), while the feed coefficient of the 40 g/kg group was significantly reduced by 9.28%. The specific growth rate and protein efficiency of the 10 g/kg group increased the most (8.27% and 13.72%, respectively). Overall, the optimal amount of EM bacteria added was 10 g/kg.

**Table 4:** Effects of the addition of an EM‐based inoculum to feed on the growth performance of *Megalobrama amblycephala*.

Group	Condition factor	Liver–body weight ratio (%)	Weight gain rate (%)	Feed coefficient (%)	Specific growth rate (%/d)	Protein efficiency (%/d)
EM0	2.89 ± 0.18^a^	1.63 ± 0.20^a^	130.30 ± 7.14^a^	112.26 ± 8.21^a^	1.45 ± 0.06	3.28 ± 0.18
EM5	2.71 ± 0.05^b^	1.76 ± 0.10^a^	138.59 ± 14.95^b^	102.49 ± 4.81^b^	1.51 ± 0.05	3.44 ± 0.16
EM10	2.83 ± 0.06^a^	1.79 ± 0.13^a^	139.86 ± 5.37^b^	104.42 ± 6.26^b^	1.57 ± 0.07	3.73 ± 0.25
EM20	2.73 ± 0.03^a^	2.05 ± 0.21^b^	139.81 ± 8.77^b^	113.47 ± 10.92^b^	1.50 ± 0.05	3.41 ± 0.16
EM40	2.76 ± 0.07^a^	1.79 ± 0.09^a^	136.88 ± 7.86^b^	101.84 ± 7.85^b^	1.48 ± 0.07	3.41 ± 0.23

*Note:* The values are presented as the means ± standard errors (SEs) of three replicates. Means in the same column with different superscripts are significantly different (*p* < 0.05). Values followed by different lowercase letters within the same list are significantly different.

^∗^ *p* < 0.05.

^∗∗^ *p* < 0.01.

### 3.2. Activities of Digestive Enzymes

Adding EM bacteria to the feed significantly affected the digestive enzyme activity of *M. amblycephala* (Table 5). The activity of gastrointestinal proteases in each experimental group increased to varying degrees, with the 10 g/kg EM group exhibiting the highest activity, which was 59.00% (*p* < 0.05) higher than that in the control group. The trend in lipase activity was similar, with the largest increase observed in the 10 g/kg EM group (54.00%, *p* < 0.05). Although a significant difference in amylase activity was not observed among the experimental groups (*p* > 0.05), amylase activity was highest in the 10 g/kg EM group.

**Table 5:** Effects of the addition of an EM‐based inoculum to feed on digestive enzyme activity in the intestine of *Megalobrama amblycephala*.

Group	Proteinase (U·mg^−1^ prot)	Lipase (U·g^−1^ prot)	Amylase (U·g^−1^ prot)
EM0	21.83 ± 1.99^a^	77.26 ± 5.35^a^	189.52 ± 10.34
EM5	28.21 ± 2.84^ab^	109.29 ± 10.81^bc^	180.67 ± 12.68
EM10	34.71 ± 1.89^b^	119.24 ± 11.03^c^	194.17 ± 16.04
EM20	27.28 ± 5.00^ab^	97.31 ± 9.81^abc^	181.08 ± 8.78
EM40	23.30 ± 4.14^a^	84.56 ± 8.70^ab^	179.21 ± 11.85

*Note:* The values are presented as the means ± SEs of three replicates. Means in the same column with different superscripts are significantly different (*p* < 0.05). Values followed by different lowercase letters within the same list are significantly different.

^∗^ *p* < 0.05.

^∗∗^ *p* < 0.01.

### 3.3. GH and IGF‐I Expressions

The expression of the genes encoding GH, GH receptor A (GHRA),GH receptor B (GHRB) and IGF‐I in the liver of *M. amblycephala* fed EM bacteria‐containing feed increased to varying degrees (Table 6) (*p* < 0.05). The expression levels of the GH and IGF‐I genes in the 10 g/kg EM group were significantly higher than those in the control group and the other experimental groups, a finding that was consistent with the change in the rate of increase in weight. The results indicated that the addition of 10 g/kg EMs to the feed had the greatest effect on the expression of the GH gene in the liver of *M. amblycephala*.

**Table 6:** Effect of adding an EM‐based inoculum to feed on the expression of IGF‐I and growth hormone genes in the liver of *Megalobrama amblycephala*.

Group	GH	GHRA	GHRB	IGF‐I
EM0	1.57 ± 0.37^a^	1.83 ± 0.62^a^	1.96 ± 0.66^a^	1.37 ± 0.61
EM5	1.77 ± 0.92^a^	1.98 ± 0.54^a^	2.44 ± 0.64^ab^	1.27 ± 0.38
EM10	3.66 ± 0.27^b^	3.15 ± 0.97^b^	2.90 ± 0.29^b^	1.93 ± 0.46
EM20	2.99 ± 0.91^c^	2.33 ± 0.54^c^	1.96 ± 0.36^a^	1.07 ± 0.41
EM40	2.39 ± 0.50^c^	2.28 ± 0.42^c^	1.61 ± 0.82^a^	1.07 ± 0.55

*Note:* The values are presented as the means ± SEs of three replicates. Means in the same column with different superscripts are significantly different (*p* < 0.05). Values followed by different lowercase letters within the same list are significantly different.

^∗^ *p* < 0.05.

^∗∗^ *p* < 0.01.

### 3.4. Lipid Metabolism in the Liver

Adding EM bacteria to the feed significantly influenced lipid metabolism in the liver of *M. amblycephala* (Table 7). As given in the table, the liver TG levels in the 5 g/kg EM group were lower than those in the control group, but the difference was not significant (*p* > 0.05). The CH contents in the livers of the 5 g/kg EM group, 20 g/kg EM group and 40 g/kg EM group were lower than that in the control group, but the difference was not significant (*p* > 0.05). The low‐density protein content in each experimental group was significantly lower than that in the control group, with the levels in the 10 g/kg EM group and 20 g/kg EM group decreasing by 63.69% (*p* < 0.05). Therefore, the addition of EMs to feed significantly reduced the low‐density lipoprotein content and significantly increased the high‐density lipoprotein content in the liver of *M. amblycephala*. Significant effects on TG or total CH (TC) levels were not observed.

**Table 7:** Effect of adding an EM‐based inoculum to feed on lipid metabolism in the liver of *Megalobrama amblycephala*.

Group	Triglyceride (mmol· mg^−1^ prot)	Cholesterol (mmol· mg^−1^ prot)	Low‐density lipoprotein (mmol·mg^−1^ prot)	High‐density lipoprotein (mmol·mg^−1^ prot)
EM0	22.10 ± 1.07	48.01 ± 10.40	33.38 ± 6.41^a^	4.10 ± 0.71^a^
EM5	20.40 ± 0.60	42.02 ± 1.50	21.64 ± 2.74^b^	6.20 ± 0.34^b^
EM10	25.00 ± 2.19	42.10 ± 4.10	12.97 ± 1.56^b^	6.10 ± 0.31^b^
EM20	22.90 ± 1.56	49.08 ± 7.90	14.29 ± 1.16^b^	4.80 ± 0.57^a^
EM40	23.00 ± 2.04	41.12 ± 2.40	12.12 ± 2.61^b^	4.70 ± 0.35^a^

*Note:* The values are presented as the means ± SEs of three replicates. Means in the same column with different superscripts are significantly different (*p* < 0.05). Values followed by different lowercase letters within the same list are significantly different.

^∗^ *p* < 0.05.

^∗∗^ *p* < 0.01.

## 4. Discussion

In this study, the addition of EMs to the feed significantly altered the liver–body ratio and condition of *M. amblycephala*. The weight gain rate, specific growth rate and protein efficiency all peaked when the dietary EM concentration reached 10 mg/kg, although no significant differences were observed in the specific growth rate or protein efficiency. These results clearly indicate that the addition of EM bacteria to feed can promote the growth of fish [24–26]. The EMs used in this study were composite bacteria. These bacteria can promote growth through the following synergistic mechanisms: (1) they secrete metabolic enzymes such as phytase and glycoside hydrolase and increase the efficiency of the decomposition of complex carbohydrates in feed [27]; (2) they can adjust the structure of the intestinal flora and reduce the abundance of pathogenic bacteria through biological hypoxia and metabolite inhibition (such as *Paenibacillus polymyxa*, which produces antimicrobial peptides) [28–33] and (3) they can improve intestinal barrier function and promote the efficient absorption of nutrients. This finding was supported by the fact that growth is also inhibited when excessive amounts of EM bacteria are added. Xu [10] reported that the addition of EM bacteria to feed can promote the growth of tilapia, but growth decreased in the high‐dose group. This result is justifiable since similar results were also observed in other aquatic species [34, 35]. The excessive addition of EM bacteria can lead to a decrease in feed intake [36]. Additionally, in this study, an increase in the liver‐to‐body ratio was negatively correlated with a decrease in condition, which indicates that EMs might preferentially promote liver metabolic function rather than body fat deposition by adjusting the energy distribution mode.

The activities of digestive and absorptive enzymes can directly reflect the digestive and absorptive capabilities of fish [37, 38], which, to some extent, determine their ability to obtain nutrients from food [39]. In stomachless fish (like most Cyprinidae species), the intestine plays a crucial role in the digestion and absorption of nutrients [40, 41], consequently affecting growth performance and feed utilisation. As the amount of EM bacteria added increased from 0 to 10 mg/kg, the intestinal activities of lipase, protease and amylase in blunt snout bream significantly increased, and previous studies have shown that *Bacillus velezensis* and *Paenibacillus polymyxa* secrete protease, lipase and complex carbohydrate‐decomposing enzymes (such as pectinase and phytase), respectively [42–44]; these bacteria directly participate in the predigestion of feed nutrients. The organic acids (pH 5.4–6.1) produced by *Lactobacillus plantarum* optimise the digestive environment through two mechanisms: meeting the activity requirements of endogenous enzymes such as pepsin [45, 46] and efficiently stimulating intestinal peristalsis and promoting enzyme–substrate contact [47]. However, as the amount of EM bacteria added further increases, intestinal activity significantly decreases, which corresponds to growth performance indicators. Additional molecular investigations are needed to elucidate the underlying mechanism [48].

GH is important for regulating the growth, development, reproduction and immunity of fish, which involve physiological processes such as regulating the nutrient distribution and promoting protein synthesis, lipid metabolism, reproduction and growth [49, 50]. This study revealed that the expression levels of key genes involved in GH signalling in *M. amblycephala* liver were significantly increased after the addition of EMs to the feed. The mRNA expression of GH, GHRA and GH receptor binding protein was highest in the 10 g/kg EM group. These findings indicate that EM bacteria regulate the growth of the body through the GH–GHR–IGF signalling pathway. GH, the core hormone of the growth axis, activates the downstream signalling cascade by binding to GHR and then inducing the liver to synthesise IGF‐I, which promotes growth [51–53]. In this study, the simultaneous upregulation of GH and GHR (5 g/kg EM group) confirmed their synergistic effects on signal transduction [54, 55].

Another focus of this study was to determine whether EM bacteria can promote growth and facilitate an increase in CH levels in the fish liver. In this study, the addition of EMs to the feed did not significantly change the TC or TG levels in the liver of *M. amblycephala* but did significantly increase the high‐density lipoprotein level and decrease the low‐density lipoprotein level. These findings indicate that EM bacteria can improve the steady state of lipid metabolism by regulating the CH reverse transport system. Previous research has shown that the functional flora of EM (*Lactobacillus fermentum*, *Lactococcus* sp. and *Lactobacillus plantarum*) can achieve metabolic regulation through two ways [56, 57]: (1) the bile salt metabolic pathway—these strains secrete bile salt hydrolase (BSH) to hydrolyse and bind bile salt, which promotes the coprecipitation of CH and free cholic acid and accelerates bile salt excretion [58, 59], and (2) the inhibition of CH synthesis—*Lactobacillus plantarum* DMDL 9010 decreases the activity of HMGCR, which is the rate‐limiting enzyme of CH synthesis, thus affecting the production of CH and reducing the CH content in serum [60–62]. Thus, the addition of EM bacteria to feed plays a beneficial role in protecting the fish liver and alleviating pathological changes [63–65].

## 5. Conclusion

Our data indicated that the addition of EMs to the feed can promote the growth of fish, significantly increasing protease and lipase activities in the intestine of *M. amblycephala*, significantly increasing the expression levels of key genes involved in GH signalling in the *M. amblycephala* liver.

## Funding

This research was supported by the National Modern Agriculture Industry Technology System (Grant CARS‐45‐20) and the Central Public‐interest Scientific Institution Basal Research Fund, Chinese Academy of Fishery Sciences, CAFS (Grant 2023TD67).

## Conflicts of Interest

The authors declare no conflicts of interest.

## Data Availability

The data that support the findings of this study are available from the corresponding author upon reasonable request.
